# Effects of Perceived School Rule Enforcement on Traditional and Cyber Victimization: A Panel Study among Early Adolescents

**DOI:** 10.3390/ijerph191610218

**Published:** 2022-08-17

**Authors:** Anna Bullo, Lyne H. Zen-Ruffinen, Peter J. Schulz

**Affiliations:** 1 Faculty of Communication, Culture and Society, Università della Svizzera Italiana, 6900 Lugano, Switzerland; 2 Department of Communication and Media, Ewha Womans University, Seoul 03760, Korea

**Keywords:** adolescents, bullying, cyber, longitudinal, school rules, victimization

## Abstract

Traditional and cyber victimization can be considered similar in several respects, including the long-lasting damage done to the wellbeing of youth. However, it is important to acknowledge that they represent two clearly distinct phenomena and, as such, the impact of school rules on their development might differ. The present longitudinal study applies a multilevel model for a change approach to data resulting from a four-waves survey that followed a random sample of 101 Swiss middle school classes (N = 1500; M_age_T1 = 11.54, SD = 0.40) for a period of two school years. Findings from the analyses—which were conducted controlling for gender and economic status—showed that those students who perceive that school rules are implemented more consistently experience a slightly less steep increase in victimization online. A similar effect for traditional victimization was not found, probably because the observed change in this phenomenon was less. Considering the overall small effects found by this research, further investigation on the relation between school rule enforcement and peer victimization is recommended.

## 1. Introduction

Research on school bullying and school violence indicates that experiences of peer victimization, defined as the intentional harming of one child or young person by another [1], are regrettably common: results from a meta-analytic review on bullying show that episodes of victimizations are reported by more than one third of adolescents [2]. According to recent rankings of the Programme For International Student Assessment (PISA) [3], the situation in Switzerland is worrying: not only do Swiss 15-year-olds report the highest rates of peer harassment, but victimization experiences appear on the rise. Compared to 2015, in 2018 there was an increase in the number of teens who reported frequent physical assaults (from 2 to 7%), threats (from 3 to 7%), derision (from 11 to 13%), mean rumors (from 7 to 11%), and theft or damage of personal properties [3]. In addition, with the spread of communication technologies, in the last two decades peer victimization has largely crossed school boundaries. According to a recent systematic review [4], rates of cyberbullying victimization worldwide range from 13.99 to 57.5%. Results from research conducted on representative samples of the Swiss adolescent are in line with these data: 16% of adolescents (12–19 years old) declare that they have received offensive texts or images via smartphone or computer, 12% report the dissemination of false or offensive information on the Internet, and 23% have suffered an attempt to damage their image online [5].

The consequences of both traditional and cyber victimization can be serious and long-lasting. Specifically, bullying victimization is likely to lead to several negative health outcomes including anxiety, depression, poor general and mental health, self-injury, and substance consumption [6]. Similarly, victims of cyberbullying may experience both internalizing (e.g., anxiety, loneliness, and depression) and externalizing problems (e.g., aggressive behaviors, substance use, theft, self-harm, and rule-breaking behaviors) [7]. In both cases, victimization can even lead to suicidal ideation and suicide attempts [6,8].

Considering the severity of these outcomes, the identification of effective preventive measures is a matter of great importance. The present study focuses on the perceived enforcement of school rules, investigating its longitudinal association with trajectories of traditional and cyber victimization.

### 1.1. School Rules and Peer Victimization

According to Bronfenbrenner’s ecological systems theory [9], violent behaviors can be explained as the result of an interplay of several contextual systems. In the specific case of children and adolescent behavior, these systems are mainly constituted by family, peers, and the school environment [10]. The fact that school plays an important role is evident: not only is it the place where children and adolescents spend most of their time, but its educational purpose makes it an especially adequate place to correct dangerous and inappropriate behavior.

In most schools, students’ behavior is regulated through the definition and application of rules. Established by authority figures such as administrators and teachers, school rules define expected conduct and discipline unacceptable behavior throughout the buildings and within classrooms [11]. The implementation of school rules is considered a key variable in peer aggression prevention [12] and previous research shows how students’ perceptions of strict rule enforcement or fair and clear rules are associated with less frequent peer victimization [12,13]. On the contrary, poor regulation could facilitate and normalize bullying behavior [13,14]. Specifically, perceptions of inconsistent clarity, fairness, and discipline of rules are reported to predict higher rates of bullying [15].

Interestingly, school rules have been found to also have an impact on levels of cyber victimization. Indeed, recent studies demonstrated that school supervision and rules are effective in preventing cyberbullying offending [15,16,17,18]. This could be explained by the fact that classmates and schoolmates represent a large part of adolescents’ social contacts [19] and therefore cyberbullies are typically peers from the same school [20]: if potential aggressors perceive school regulations that condemn aggressive conduct, it is possible that peer victimization will be reduced even in online contexts.

These results are in line with the authoritative school climate theory [12], which posits that school safety is largely impacted by two key factors: support (i.e., availability of caring adults) and structure (i.e., consistent enforcement of school discipline). The present study specifically focuses on the second construct and examines its longitudinal association with traditional and cyber victimization. This investigation is important for two main reasons: first, entrance into adolescence is typically characterized by increased engagement in problematic behavior [21], which means that perceptions of school rule enforcement and rates of victimization might undergo significant transformations that cannot be detected through cross-sectional designs. Second, although similar in several respects, traditional and cyber victimization have different characteristics that might alter the effects of school rules.

### 1.2. Traditional vs. Cyber Victimization

Cyber victimization is often seen as a new form of victimization, where communication technologies represent an alternative channel for engaging in the same aggressive behaviors seen in offline environments. While past research confirms that there is often a significant overlap between traditional and cyber forms of peer harassment [22], it is important to acknowledge that they possess well-distinct characteristics. Specifically, traditional and cyber victimization can be distinguished by the *type of aggression* (unlike traditional victimization, cyber victimization does not include physical forms of aggression), the *potential for anonymity* (which is significantly higher in online settings), the *strength differential* (online attacks can be easily perpetrated also by weak and unpopular individuals), and *repeatability* (online mean messages and other harmful contents can potentially be shared an infinite number of times by different users) [23]. In addition, unlike in-person acts of aggression, cyber victimization can occur anywhere and at any time of the day [24]. These last properties not only contribute to increase the vulnerability of victims, but, together with the potential for anonymity, significantly hinder the supervision of authority figures [24,25]. This could mean that traditional and cyber victimization are differently affected by the enforcement of school rules.

Then, while school rules might contribute to reduce rates of victimization, it is also important to consider the distinct developmental patterns of these phenomena.

In the study of peer victimization, age is expected to play a key role [10]. Previous studies show how the risk of victimization is especially high during adolescent years [26]. Rates of traditional victimization are found to peak during middle school years and decline somewhat by the end of high school [27].

Systematic reviews suggest that cyber victimization frequency follows a similar developmental pattern, increasing during the middle school years and declining thereafter [28]. However, more recent studies show how experiences of online victimization are common even among young adults [29]. Specifically, because cyberbullying perpetration is reported to steadily increase until the mid-twenties [30], it is possible that victimization remains more prevalent even in the second half of adolescence.

A further important factor that distinguishes traditional and cyber victimization is gender. Previous research shows that direct forms of aggression, such as physical harassment and overt verbal attacks, are significantly more common among boys [31], who are also more often victims of these behaviors [32]. Then, although gender differences in the perpetration of indirect forms of aggression (e.g., social exclusion and gossiping) are reported to be trivial, girls appear to be more often victims of it [32]. While the phenomenon of victimization includes both direct and indirect forms of aggression, past research suggests that boys are more often victims of traditional bullying than girls [33,34]. However, when victimization takes place online, the role of gender no longer seems decisive: various researchers report no significant gender difference for cyberbullying victimization [7,25,28,35,36]. However, according to other studies, girls seem to have a significantly higher likelihood of being cyberbullied [37,38], especially when bullying is perpetrated through social networking websites [39], emails [25], phone calls, and text messages [38].

Finally, another aspect that might differently affect traditional and cyber victimization is socio-economic status (SES). Findings from empirical studies show that children coming from a socioeconomically disadvantaged family (low SES) have an increased risk of being involved in bullying [40,41,42]. This relation could be explained by the fact that lower parental educational level typically reflects their problem-solving skills, intellectual resources, literacy, norms, and values, as well as general and specific knowledge [40]. All these factors might influence children’s behavior, including their coping strategies and social skills.

For what concerns cyber victimization, the association with SES is far less clear. While some studies report that low SES is associated with a greater risk of being cyberbullied [43,44], others show that high SES adolescents are more likely to be cyberbullied [45,46]. One explanation for a positive association between SES and cyber victimization could be that communication technologies are more easily accessible to adolescents from wealthier families [45,47,48], and therefore their risk of being victimized is increased. However, further research on this topic is needed [49].

### 1.3. The Current Study

Traditional and cyber victimization are both severe threats to the wellbeing of adolescents and identifying effective strategies to reduce them is a matter of great importance.

The main aim of the present research is therefore to investigate whether a consistent implementation of school rules might help reduce these behaviors. Because both types of victimization and perceptions of school rule enforcement might change over time, a longitudinal approach is adopted. More specifically, the frequency of victimization and perceived school rule enforcement will be observed in four occasions during the first two years of middle school. Considering that previous studies on age effects identified growing rates of traditional and cyber aggression throughout adolescent years [27,28,30], it was hypothesized that experiences of traditional and cyber aggression would increase over time (H1). Then, based on the assumptions of the authoritative school climate theory [12], it was further hypothesized that trajectories of traditional and cyber victimization would be influenced by the presence of consistent school rules, so that those adolescents who report that their school implements rules more consistently would experience less frequent victimization (H2a). However, because cyber victimization is characterized by higher elusiveness [24,25], it was also hypothesized that the association between perceived enforcement of school rules and cyber victimization would be weaker when compared to the association of school rule enforcement and traditional victimization (H2b). Finally, the impact of school rules on the development of peer aggression is controlled for two relevant sociodemographic factors: gender and perceived economic status. Particularly, because evidence from previous research suggests that traditional victimization is typically higher among boys [33,34], it was hypothesized that male adolescents would experience higher initial rates and a steeper increase as compared to girls (H3a). Then, because most research indicates that gender does not play a significant role in online victimization [7,28], it was expected that boys and girls would report a similar frequency of cyber victimization (H3b). As previous studies show a negative relation between SES and traditional victimization, it was further hypothesized that adolescents reporting lower perceived family income would show a higher risk of victimization (H4a). Because adolescents from wealthier families might receive their own smartphone or computer at a younger age, it was expected that they would report a higher initial status of online victimization (H4b).

## 2. Materials and Methods

### 2.1. Participants and Procedure

To conduct the present research, data from a panel survey were used. The survey is part of AReS, a longitudinal project conducted between 2017 and 2021 in the middle schools of the Canton of Ticino, Switzerland. Participants to the panel survey were randomly selected, and constitute a representative sample of the early adolescent population of the Canton (N_T1_ = 2052). Details about the sampling procedure, survey administration, and privacy guarantee are provided elsewhere [50].

The sample for this study includes 1508 students from public middle schools who were followed from the beginning of their first year (Fall 2017) to the end of their second (Spring 2019) through four questionnaires administered at six-month intervals. Of the 2052 students who were invited to participate at T1, 2022 participated at T1, 1879 at T2, 1896 at T3 and 1865 at T4. Most missing data are due to absences the day of data collection or students moving outside the Canton; less than 4% of the students (N = 75) did not participate because their parents objected. To be included in the sample, it was required that students participated at least in three waves, that they were part of classes where no error in the administration of surveys took place, and that their answers were deemed reliable. Answers were considered unreliable in the presence of (a) evident patterns in the answers (e.g., answers’ crosses arranged in a zigzag), (b) multiple contradictory answers, (c) multiple unlikely answers, and (d) explicit declarations of untruthful answers. This procedure led to a final sample of 1508 students.

### 2.2. Measures

#### 2.2.1. Traditional Victimization

Traditional victimization was measured using six items selected from the European Bullying Intervention Project Questionnaire (EBIPQ) [51]. Specifically, participants were asked to indicate how frequently they had been victims of physical aggression (e.g., being hit, kicked, or pushed), direct verbal harassment (e.g., being insulted or threatened) and relational forms of aggression (e.g., being excluded or being a victim of badmouthing and gossiping) using a 4-point response scale that went from “Never” (1) to “Often” (4). Cronbach’s alpha for the six items was 0.821 at T1, 0.846 at T2, 0.824 at T3, and 0.840 at T4.

#### 2.2.2. Cyber Victimization

Cyber victimization was measured using six items selected from the European Cyberbullying Intervention Project Questionnaire (ECIPQ) [52]. In this case participants were asked to report how often they had been victims of online forms of aggression including direct verbal aggression (e.g., being insulted via texts or online messages; being threatened), relational aggression (e.g., being excluded online; being a victim of badmouthing or gossiping) and media-related aggression (e.g., unwanted sharing of embarrassing pictures or videos). As with traditional victimization, the answer scale went from “Never” (1) to “Often” (4). Cronbach’s alpha for the six items was 0.839 at T1, 0.828 at T2, 0.856 at T3 and 0.873 at T4.

#### 2.2.3. School Rule Enforcement

The construct of school rule enforcement aims to determine the consistency with which infringements of regulation are sanctioned. Specifically, the construct concerns students’ *perceptions* of school rule enforcement: it is indeed expected that adolescents’ beliefs about the presence or absence of consistent rules will be strictly related to their own behavior. The measure included eight items and participants were asked whether in their school there are negative consequences for those students who are caught committing the following behaviors: such as (1) alcohol drinking, (2) smoking, (3) insulting or teasing a schoolmate, (4) pushing or hitting a schoolmate, (5) excluding a schoolmate, (6) showing disrespect towards teachers, (7) using bad language, and (8) using mobile phones when not permitted. The answer scale went from “No, never” (1) to “Yes, always” (5).

It was verified that the problematic behaviors included in the items were part of school regulations by asking students whether in their school there are rules that prohibit that type of conduct. Results obtained at T1 show that over 95% of students report that smoking, alcohol consumption, physical aggression, disrespect of teachers, and use of mobile phones are forbidden behaviors within their school. Slightly lower percentages were reported for verbal violence (93%), use of bad language (84%), and exclusion (71%). These results are also in line with findings of a content analysis conducted on written regulations and policies, which showed how 97% of participating schools have written rules that demand good conduct, which includes respect of schoolmates and teachers (89%), no smoking (89%) and alcohol drinking (70%), no use of physical (78%) and verbal violence (78%), and appropriate use of mobile phones (95%) [53].

The construct of perceived school rule enforcement was assessed at all four measurement occasions, and Cronbach’s alpha for the eight items was 0.916 at T1, 0.847 at T2, 0.878 at T3, and 0.849 at T4.

#### 2.2.4. Sociodemographic Variables

Assessed sociodemographic variables include gender (male or female), age (computed from birthdate and dates of data collection) and perceived economic status of the family. Concerning this last variable, participants were asked at T1 to indicate how their family is doing financially using a scale that went from “Not well at all” (1) to “Very well” (5).

### 2.3. Data Analysis

#### 2.3.1. Missing Data

Missing data related to the variables of interest are below the recommended threshold of 5% [54], ranging between 1.9 and 4.4%. Little’s missing completely at random test indicated that data in the scales included in the analyses were possibly missing at random χ^2^ (73, N = 1508) = 52.388, *p* = 0.967. Although estimation of missing data is not necessary for missing rates below 5% [54], the remaining missing values were imputed using a Bayesian regression imputation method and a predictive mean matching model. Results emerging from imputed data did not differ from those obtained with non-imputed data, neither in terms of effect size, nor in significance. For simplicity, below we refer to the analyses conducted on original data (not imputed). Because outliers might bias the estimator, eight and nine cases were removed from the non-imputed and the imputed dataset, respectively. Although the deletion of outliers did not affect the results, it was preferred to keep the solution that is closer to the unbiased estimator (i.e., without the outliers).

#### 2.3.2. Multilevel Model for Change

Trajectories of traditional and cyber victimization were analyzed using growth models within a multilevel modeling approach [55]. While multilevel analysis is typically used to study the effects of different levels of aggregation, such as classes and schools, in the multilevel model for change approach, levels correspond to within-person (Level 1) and between-person (Level 2) changes. More specifically, Level 1 deals with the individual change that each subject is expected to experience during the period of observation. In this study, this corresponds to the 2-year change in traditional and cyber victimization experienced by each adolescent. Level 2 corresponds to changes attributable to influence factors that characterize groups of individuals within a sample. The influence factors considered in this study are sociodemographic variables (i.e., gender and economic status), and school rule enforcement. The two-level hierarchical models were estimated using a Maximum Likelihood method in SPSS Statistics 25. In total, five models were calculated. Model A is the unconditional mean model and indicates if there is systematic variation in the dependent variable that is worth exploring. Model B is the unconditional growth model, which adds a parameter of time (in this case participants’ age). Model C, D, and E introduce predictive factors (i.e., gender, economic status, and school rule enforcement) one at a time. All predicting variables were mean centered. To make the intercept more meaningful, the age variable was computed by subtracting the mean age at T1.

## 3. Results

### 3.1. Descriptive Results

The 1500 analyzed subjects have a mean age of 11.54 (SD *=* 0.40) at T1 and 13.04 (SD = 0.40) at T4. Girls make up 48% of the sample. The descriptive statistics for victimization and school rule enforcement are reported in Table 1. For what concerns traditional and cyber victimization, it can be observed that mean values are very low, as they are quite close to one, which is the minimum value of the scale used. However, as shown in Table 2, there is a quite considerable percentage of students who report having been subjected to acts of aggression on a fairly regular basis. For instance, on the last measurement occasion, about one in eight students report having suffered physical aggression “sometimes” or “often” in the previous six months, while frequent direct verbal violence in traditional and online contexts concerns reported by about 23% and 12% of the participants. This means that the phenomenon of victimization is present, but it concerns only a part of adolescents. In addition, it is interesting to note that levels of traditional and cyber victimization at T4 are higher than those reported at T1, suggesting an overall increasing trend. Finally, for what concerns the enforcement of school rules, mean values reported in Table 1 indicate that, in general, adolescents perceived that school rules are implemented quite often.

Table 3 shows the correlations between the main variables. Traditional and cyber victimization measured at the same occasion resulted in a strong correlation, indicating that the two phenomena often overlap. However, it is interesting to note that the correlation between the perceived enforcement of school rules and victimization was significant only on a few occasions. More specifically, at T3 there was a weak correlation with cyber victimization (r = −0.057, *p* < 0.05), while at T4 there was a weak correlation with both traditional and cyber victimization (r = −0.096, *p* < 0.01 and r = −0.107, *p* < 0.01, respectively). At T1, the correlation of victimization with perceived socio-economic status was also significant but weak. In addition, Table 3 shows that perceptions of school rule enforcements are not very stable, as the auto-correlation is quite low, especially between T1 and T2 (r = 0.257, *p* < 0.05). However, it is interesting to note that the magnitude of this auto-correlation increases over time.

### 3.2. Multilevel Model for Change

#### 3.2.1. Age

As illustrated by Model B (the unconditional growth model) in Table 4, participants’ age was significantly associated with values of traditional victimization, so that older adolescents are slightly more likely to report victimization (0.05, SE = 0.01; *p* < 0.001). During the two years of study, the total increase in traditional victimization was therefore of 0.15 points (3.75%). Adding the time factor to the model contributes to the decrease of both the within-person variance and the between-subjects variance in the initial status. The co-variance component is non-significant, meaning that there is no significant relation between the initial status and the rate of change (i.e., the frequency of victimization does not grow faster for those with lower initial levels of victimization). For what concerns cyber victimization, results from the unconditional growth model (Table 5) show that the frequency of being victimized increased by 0.10 points every six months (SE = 0.01, *p* < 0.001). The overall growth is therefore double that of traditional aggression (7.5%). Additionally in this case, adding the time factor to the model decreases the within-person variance and the between-subjects variance in the initial status. In the latter case, the variance component is halved, meaning that the variable of age helps to explain differences between subjects. The hypothesis predicting that experiences of traditional and cyber aggression would increase over time (H1) was therefore confirmed.

#### 3.2.2. Gender

Results indicate that gender is significantly associated with the initial status of traditional victimization. More specifically, being a girl reduces by 0.09 (SE = 0.03, *p* < 0.001) the frequency of victimization. Although traditional victimization increases over time, gender was not found to have a significant impact on the rate of change. Hypothesis H3a was therefore only partially confirmed. Interestingly, adding gender to the model does not contribute to reducing the variance components, meaning that differences within and between individuals are explained by other factors. In the case of cyber victimization, results show that gender does not play a significant role, neither for the initial status, nor for the rate of change. The hypothesis H3b was therefore confirmed.

#### 3.2.3. Perceived Economic Status

Similarly to gender, perceived economic status was found to be significantly associated with the initial status of traditional victimization, but not with its rate of change. More specifically, individuals who perceived a better economic status of their family reported slightly lower initial levels of victimization (−0.06, SE = 0.02; *p* < 0.001). Hypothesis H4a was therefore confirmed. Additionally in this case, adding the economic status variable to the model did not reduce the remaining variance. For what concerns cyber victimization, no significant associations were found. Hypothesis H4b was therefore not confirmed.

#### 3.2.4. School Rule Enforcement

Unlike gender and perceived economic status, the perceived enforcement of school rules was not significantly associated with traditional victimization. More specifically, those subjects who reported consistent school rules were not less likely to be victimized compared to those who perceived that rules are enforced less often, nor did they experience a less steep increase in victimization. Again, there was not a change in the predicted variance components.

In the case of cyber victimization, the association with the initial status remained non-significant; however, the association with the rate of change was significant. Indeed, results indicate that those adolescents who perceive that school rules are implemented more often experience a slightly less steep increase in cyber victimization (−0.02, SE = 0.01; *p* < 0.05). Adding this variable to the model contributes to slightly reduce the between-subjects variance in the rate of change. The hypothesis that predicted a negative association between perceived school rule enforcement and victimization (H2a) was therefore only partially confirmed for cyber victimization. In addition, while hypothesis H2b predicted that the role of school rules would be more relevant in the case of traditional victimization, obtained results show that they have a significant impact only on the cyber one.

## 4. Discussion

The main aim of the present research was to investigate the effectiveness of perceived school rule enforcement with regard to the development of traditional and cyber victimization.

Recent studies conducted in Switzerland suggest that cases of frequent victimization in schools are on the rise [3,5]. While the results of our analyses show that a large part of our participants do not seem to be affected by this issue, it is important to recognize that there is a lower, but still considerable, number of adolescents who report fairly frequent episodes of victimization.

In addition, obtained results show that as adolescents grow up, the frequency of victimization tends to grow as well. Although the found effect is quite small, this finding is in line with previous research, according to which experiences of victimization increase with age [27,28,30].

In addition, it is interesting to note that the rate of change in cyber victimization experiences is higher than that of traditional victimization. This result could be explained by the fact that with entry into adolescence, the preferred modes of aggression progressively change. More specifically, physical aggression (which constitutes an important dimension of traditional victimization) is reported to decrease as children enter adolescent years [56]. Moreover, it is also important to mention that the beginning of adolescence typically coincides with an increase in time spent online. Specifically, in Switzerland, kids receive their first smartphone between the ages of 10 and 13 [57] and are online for about two hours on weekdays and three hours on weekends [58]. Because ownership of a personal device hinders parental surveillance [59], the risk of victimization might significantly increase.

Before examining the impact of school rules, it was verified whether the socio-demographic variables of gender and economic status determined different trajectories of victimization. Specifically, it was found that girls and adolescents from wealthier families are less likely to be victims of traditional aggression. For what concerns the role of gender, this finding is not surprising: traditional forms of aggression, and especially those of a physical nature, are consistently reported to be more common among boys. Although some researchers reported that girls might be at higher risk of victimization perpetrated online, in line with a broader body of research, results of this study show no significant gender difference. Considering the young age of our sample this result is not surprising: gender disparities in victimization might indeed emerge only as adolescents grow older. Particularly, in the second half of adolescence, online sexual harassment increases considerably [58] and, in this case, girls are often the main target. Swiss data on sexual assault on children and adolescents indicate that almost every third girl experienced sexual harassment via electronic media, while among boys this is experienced by around one in ten [60].

Then, regarding the perceived economic state, results showed that adolescents who reported a better economic situation were also less likely to be victims of traditional aggression. Interestingly, this protective effect was not found for cyber victimization. This could be explained by the fact that an online environment has an equalizing effect, so that even individuals with a higher social status can be attacked [23]. Previous findings suggest that a higher SES might be associated with higher cyber victimization experiences, as greater economic availability would allow easier access to communication technologies [45,47,48]. However, this did not turn out to be the case, probably because in Switzerland economic disparities are not that great and even the poorest families can afford internet-enabled devices.

Then, after controlling for the impact of gender and economic status, the role of school rules was analyzed. Although one would hope that perceptions of consistent school rule enforcement help to decrease the occurrence of victimization, results of this research reveal that their association with the development of traditional victimization is minimal. Indeed, the perceived implementation of school rules was scarcely correlated with victimization rates. Our growth analyses also showed a significant association only with the rate of change in cyber victimization, meaning that in schools promoting a more secure environment, adolescents experience a less steep increase in victimization. While the found effect was very small and needs further research to be confirmed, the result remains rather surprising. Compared to online victimization, traditional victimization is in fact a phenomenon much more linked to the school context. For this reason, it was hypothesized that the role of school rules be more relevant in the case of traditional aggression. The found result might be explained by the fact that the observed change in cyber victimization was greater than that observed for traditional victimization. If the change in traditional victimization were as great, maybe it would have been possible to identify a similar effect.

A further important consideration to make is that regulations considered in this study were not specific to traditional and cyber victimization, but included more general rules about problematic behaviors, such as aggression, but also substance consumption and disrespectful conduct. While more specific rules might have shown a greater impact on victimization rates, it is interesting to note that the simple fact of providing a structured environment that discourages deviant behavior is beneficial in slowing down—albeit slightly—the insurgence of cyber victimization. Considering the small effects found by this research, it is nevertheless important to conduct further studies on this issue.

Specifically, future research should consider that, in this study, the focus was on perceptions of school rule enforcement, which might differ from actual enforcement. In terms of interventions, this constitutes a double challenge. First, it means that schools do not only need to implement their rules in a consistent way, but also that this implementation is somehow visible to the students. This does not mean that sanctioning should necessarily become public, but that even students who have not yet engaged in a prohibited behavior are well aware of the negative consequences they might incur in case of transgression. Second, perceptions are reconstructions of reality based on individual observations and experiences, meaning that specific episodes and memorable events might significantly modify them. This might possibly explain why, in this study, it was found that students perceptions of school rule enforcement are not stable over time: as adolescents witness an increasing number of transgressions and sanctions, their perceptions of rule enforcement might be consequently adapted. For instance, when starting middle school, a student might believe that the consequences for insulting another student are severe. However, over time, she/he realizes that this type of behavior is quite common among schoolmates and that teachers rarely intervene, therefore his/her perception is modified accordingly. Future studies should therefore investigate more thoroughly the association of perceived rule enforcement and exposure to cases of transgression.

This type of investigation would also be needed to better clarify the direction of causal effects. Indeed, while the authoritative school climate theory posits that the consistent enforcement of school discipline can positively impact students’ behavior, it is true that the opposite might be true as well. More specifically, the high diffusion of problematic conduct among students might lead teachers and other school authorities to react and implement regulations more strictly. Or, conversely, teachers who are often confronted with rule infringements might end up with becoming more tolerant, as adequately punishing every transgression might be too time consuming. This last scenario might generate a downward spiral, where inconsistent sanctioning leads to further cases of problematic behaviors, including peer victimization. The possibility of a reciprocal influence of school rule enforcement and misconduct further stresses the need for longitudinal designs that permit to establish the sequence of events.

### Limitations and Implications for Future Studies

A first important limitation of this study concerns the low rates of victimization reported by participants. In fact, most subjects did not report any episode of victimization. While the scarce diffusion of peer aggression is certainly a positive thing, it is true that low scores might also be the result of a social desirability bias. Particularly, it might be that adolescents are reluctant to identify themselves as victims. This could mean that victimization in schools is actually more common than emerges from this study. However, the administered survey also investigated other sensitive topics, including self-harm. Because the reported frequency of this behavior was higher than expected, there is reason to believe that participants were not particularly afraid to disclose personal information (most likely because of the anonymity guarantee). This could mean that rates of aggression are indeed quite low, and the use of a more sensitive scale could have helped to find greater variance in this construct. A further explanation for the low rates of victimization could be attributed to difficulties in remembering all victimization episodes. Additionally in this case, a more detailed answer scale might have helped to increase variation in answers.

A second limitation of this study concerns the measure used to assess the implementation of school rules, which was not specific for aggression, but also included other problematic behaviors. While this aspect highlights the importance of a structured school context, it is true that investigating the effects of more specific rules might have revealed a greater impact on the development of aggressive conduct. In addition, this measure was based on student’s *perceptions* of rules implementation. While it is true that individuals typically act on the basis of their subjective interpretations [61], it might be that their perceptions do not correspond to reality. It might therefore be interesting to verify whether more objective measures (e.g., teachers’ reports of transgressions and sanctions) would provide similar results.

A further limit is then related to measurement of the economic status. Additionally in this case, the measure was based on students’ perceptions, which might have led to inaccurate estimates of this construct: young adolescents may indeed not be fully aware of their family’s economic situation. In addition, unlike all the other measures, the perceived economic status was assessed only at T1. Although it can be assumed that large economic changes in such a short period of time are rare (Switzerland is a fairly stable country from an economic point of view), the assessment at different points in time could have returned a more detailed view of the role of economic status.

Then, it should be noted that, despite the longitudinal nature of the study, the analyses conducted do not allow causal conclusions to be drawn: rule enforcement and other statistical techniques (e.g., covariance structure analysis) might have been more informative about the directionality of effects.

Finally, future studies might further explore the effectiveness of school rule enforcement by also considering students’ perpetration of aggressive behaviors. The behavior of bullies is indeed another important aspect that should be taken into consideration in the prevention of victimization.

## 5. Conclusions

The present study compares the development of traditional and cyber victimization among a sample of Swiss early adolescents and explores their longitudinal association with perceptions of school rules implementation. Interestingly, findings reveal a small effect only in the case of cyber victimization. More specifically, it was found that those students who perceive that school rules are implemented more consistently experience a slightly less steep increase in victimization online. This result is particularly surprising, as school rules would be expected to be less effective in the case of aggressive behaviors that can easily take place outside the school boundaries or be perpetrated anonymously. Further research on the effects of school rules on traditional and online aggression is therefore encouraged.

## Figures and Tables

**Table 1:** Descriptive Statistics: Longitudinal assessment of traditional victimization, cyber victimization and perceived school rule enforcement.

	Waves			
	T1	T2	T3	T4
Traditional victimization				
N	1465	1434	1472	1434
Mean (SE)	1.41 (0.01)	1.51 (0.02)	1.49 (0.01)	1.49 (0.02)
Skewness (SE)	1.81 (0.06)	1.44 (0.07)	1.55 (0.06)	1.48 (0.07)
Kurtosis (SE)	4.19 (0.13)	1.88 (0.13)	3.00 (0.13)	2.04 (0.13)
Cyber victimization				
N	1455	1436	1453	1433
Mean (SE)	1.15 (0.01)	1.29 (0.01)	1.20 (0.01)	1.35 (0.01)
Skewness (SE)	3.51 (0.06)	2.02 (0.07)	3.18 (0.06)	2.01 (0.07)
Kurtosis (SE)	17.11 (0.13)	4.47 (0.13)	13.36 (0.13)	4.24 (0.13)
School rule enforcement				
N	1461	1442	1469	1440
Mean (SE)	3.75 (0.03)	3.92 (0.02)	3.82 (0.02)	3.81 (0.02)
Skewness (SE)	−0.90 (0.06)	−1.03 (0.06)	−0.96 (0.06)	−0.57 (0.06)
Kurtosis (SE)	−0.08 (0.13)	0.90 (0.13)	0.62 (0.13)	−0.11 (0.13)

**Table 2:** Percentages of adolescents reporting different types of victimization. Frequent victimization (“sometimes” or “often”) is reported in brackets.

	T1			T2			T3			T4		
	Girls (n = 718)	Boys (n = 782)	Tot. (n = 1500)	Girls (n = 718)	Boys (n = 782)	Tot. (n = 1500)	Girls (n = 718)	Boys (n = 782)	Tot. (n = 1500)	Girls (n = 718)	Boys (n = 782)	Tot. (n = 1500)
Traditional victimization												
Physical aggression	27.2% (4.3%)	46% (10.6%)	36.9% (7.5%)	33.4% (10.5%)	49.2% (16.7%)	41.7% (13.8%)	28.6% (4.7%)	47.2% (13.2%)	38.2% (9.1%)	25.5% (8.4%)	42.6% (16.6%)	34.3% (12.6%)
Insults	44.1% (8.7%)	58.5% (16.8%)	51.6% (12.9%)	46.5% (20%)	61.1% (28.4%)	54.1 (24.4%)	49.9% (14.1%)	66.1% (23.1%)	58.3% (18.8%)	50% (20.7%)	60.6% (25.7%)	55.5% (23.3%)
Badmouthing	33.9% (8%)	33.2% (8.3%)	33.6 % (8.2%)	35.8% (15.8%)	32.2% (11.4%)	33.9% (13.5%)	38.7% (12.5%)	40% (12.2%)	39.4% (12.4%)	36.8% (15%)	31.9% (10.9%)	34.3% (12.9%)
Threats	10.5% (2.4%)	16.8% (6%)	13.8% (4.3 %)	12.8% (3.4%)	20.9% (7.1%)	17% (5.3%)	11.6% (3%)	20.5% (5.5%)	16.2 % (4.3%)	10% (2.6%)	21.1% (6.9%)	15.7% (4.8%)
Exclusion	30.8% (5.7%)	30.3% (6.1%)	30.5% (5.9%)	38.2% (14.9%)	34.1% (10.9%)	36.1% (12.9%)	37.4% (8.5%)	32.1% (7.9%)	34.6% (8.2%)	35.5% (14.9%)	29.7% (9.5%)	32.5% (12.1%)
Gossip	22.8% (6.9%)	23.2% (5%)	23% (5.9%)	25.8% (8.8%)	23.3% (7.4%)	24.5% (8.1%)	31.1% (10.5%)	29.3% (8.9%)	30.1% (9.6%)	28.9% (10.9%)	24% (7.5%)	26.4% (9.1%)
Cyber victimization												
Insults	16.4% (3.3%)	18.7% (2.7%)	17.6% (3%)	29.9% (11.2%)	31.5% (11.6%)	30.7% (11.4%)	19.2% (3.9%)	24.6% (4.8%)	22% (4.4%)	29.5% (10.3%)	35.5% (13.8%)	32.6% (12.1%)
Badmouthing	18.3% (3.9%)	17.2% (2.3%)	17.7% (3%)	34.7% (14.2%)	29.9% (9.5%)	32.2% (11.7%)	23.2% (4.4%)	20% (5.6%)	21.5 % (5%)	34.7% (13.6%)	33.2% (11.3%)	33.9% (12.4%)
Threats	5.6% (1.3%)	9.8% (2.4%)	7.7% (1.8%)	11.4% (3.6%)	15.2% (4.3%)	13.4% (4%)	4.9% (1%)	12% (3.6%)	8.5% (2.3%)	8.9% (2.9%)	19.5% (6.4%)	14.4% (4.8%)
Sharing of embarrassing pictures/videos	2.7% (0.6%)	4.4% (0.9%)	3.5% (0.7%)	5.5% (2.2%)	7.2% (2%)	6.4% (2.1%)	4.3% (1%)	6% (1.9%)	5.2% (1.5%)	6.6% (2%)	14.7% (5%)	10.8% (3.6%)
Exclusion	13.8% (2.3%)	15.2% (2.9%)	14.5% (2.6%)	21.3% (5.4%)	19.6% (5.1%)	20.5% (5.3%)	18.5% (3.3%)	18% (3.2%)	18.2% (3.2%)	25.7% (8%)	25.2% (7.8%)	25.4% (7.9%)
Gossip	13.2% (2.6%)	11.5% (1.6%)	12.4% (2.1%)	24.3% (7.4%)	17.5% (3.9%)	20.9% (5.7%)	18% (3.5%)	14.8% (4%)	16.3% (3.7%)	26.7% (10.8%)	25.2% (6.7%)	25.9% (8.7%)

**Table 3:** Correlations among variables of study.

Variables	1	2	3	4	5	6	7	8	9	10	11	12	13
1. TV T1	-												
2. TV T2	0.443 **	-											
3. TV T3	0.384 **	0.534 **	-										
4. TV T4	0.310 **	0.461 **	0.513 **	-									
5. CV T1	0.578 **	0.286 **	0.257 **	0.209 **	-								
6. CV T2	0.323 **	0.570 **	0.351 **	0.297 **	0.385 **	-							
7. CV T3	0.253 **	0.390 **	0.598 **	0.362 **	0.313 **	0.408 **	-						
8. CV T4	0.217 **	0.353 **	0.381 **	0.581 **	0.253 **	0.348 **	0.381 **	-					
9. SRE T1	0.023	0.008	0.015	−0.033	0.024	−0.034	−0.040	−0.013	-				
10. SRE T2	−0.026	−0.022	−0.008	0.030	−0.037	−0.042	−0.059 *	−0.045	0.287 **	-			
11. SRE T3	−0.004	−0.048	−0.013	−0.053 *	−0.019	−0.074 **	−0.057 *	−0.092 **	0.257 **	0.335 **	-		
12. SRE T4	−0.055 *	−0.106 **	−0.050	−0.096 **	−0.047	−0.104 **	−0.046	−0.107 **	0.113 **	0.267 **	0.428 **	-	
13. economic status T1	−0.129 **	−0.035	−0.067 *	−0.035	−0.071 **	−0.058 *	−0.018	−0.050	0.012	0.017	−0.021	0.011	-

Note. * Correlation is significant at the 0.05 level (2-tailed). ** Correlation is significant at the 0.01 level (2-tailed). CV = Cyber Victimization; TV = Traditional Victimization; SRE = School Rule Enforcement.

**Table 4:** Results of fitting a multilevel model for change to the traditional victimization data: fixed effects, variance components and goodness of fit.

		Model A	Model B	Model C	Model D	Model E
		Estimate (SE)	Estimate (SE)	Estimate (SE)	Estimate (SE)	Estimate (SE)
*Fixed Effects*						
Initial status	Intercept	1.48 *** (0.01)	1.44 *** (0.01)	1.48 *** (0.02)	1.48 *** (0.02)	1.48 *** (0.02)
	Gender			−0.09 *** (0.02)	−0.09 * (0.02)	−0.09 * (0.03)
	Economic status				−0.06 *** (0.02)	−0.06 *** (0.02)
	School rule enforcement					0.00 ns (0.01)
Rate of change	Intercept		0.05 *** (0.01)	0.05 * (0.01)	0.04 * (0.01)	0.04 * (0.01)
	Gender			0.02 (0.02) ns	0.02 ns (0.02)	0.02 ns (0.02)
	Economic status				0.02 ns (0.01)	0.02 ns (0.01)
	School rule enforcement					−0.01 ns (0.01)
*Variance Components*						
Level 1	Within-person	0.17 *** (0.00)	0.15 *** (0.00)	0.15 *** (0.00)	0.16 *** (0.00)	0.16 *** (0.00)
Level 2	In initial status	0.13 *** (0.01)	0.11 *** (0.01)	0.11 *** (0.01)	0.11 *** (0.01)	0.11 *** (0.01)
	In rate of change		0.03 *** (0.01)	0.03 *** (0.01)	0.03 *** (0.01)	0.03 *** (0.01)
	Co-variance		0.00 ns (0.01)	0.00 ns (0.01)	0.00 ns (0.01)	0.00 ns (0.01)
*Goodness of Fit*						
	Deviance	8270.16	7880.07	7865.92	7619.78	7549.17
	AIC	8276.16	7892.07	7881.92	7639.78	7573.17
	BIC	8296.16	7931.91	7935.04	7705.80	7652.24

Note: Cells show the unstandardized estimates and their standard deviations in brackets. * *p* < 0.05, *** *p* < 0.001, ns = not significant.

**Table 5:** Results of fitting a multilevel model for change to the cyber victimization data: fixed effects, variance components, and goodness of fit.

		Model A	Model B	Model C	Model D	Model E
		Estimate (SE)	Estimate (SE)	Estimate (SE)	Estimate (SE)	Estimate (SE)
*Fixed Effects*						
Initial status	Intercept	1.25 *** (0.01)	1.17 *** (0.01)	1.17 *** (0.01)	1.17 *** (0.01)	1.17 *** (0.01)
	Gender			0.01 ns (0.02)	0.01 ns (0.02)	0.01 ns (0.02)
	Economic status				−0.01 ns (0.01)	−0.01 ns (0.02)
	School rule enforcement					0.00 ns (0.01)
Rate of change	Intercept		0.10 *** (0.01)	0.11 *** (0.01)	0.11 *** (0.01)	0.11 *** (0.01)
	Gender			−0.03 ns (0.02)	−0.03 ns (0.02)	−0.03 ns (0.02)
	Economic status				−0.02 ns (0.01)	−0.02 ns (0.01)
	School rule enforcement					−0.02 * (0.01)
*Variance Components*						
Level 1	Within-person	0.13 *** (0.00)	0.12 *** (0.00)	0.12 *** (0.00)	0.12 *** (0.00)	0.12 *** (0.00)
Level 2	In initial status	0.06 *** (0.00)	0.03 *** (0.00)	0.03 *** (0.00)	0.03 *** (0.00)	0.03 *** (0.00)
	In rate of change		0.02 *** (0.00)	0.02 *** (0.00)	0.02 * (0.00)	0.01 * (0.00)
	Co-variance		0.02 *** (0.00)	0.02 *** (0.00)	0.02 *** (0.00)	0.02 *** (0.00)
*Goodness of Fit*						
	Deviance	6263.77	5733.11	5729.62	5532.14	5480.82
	AIC	6269.77	5745.11	5745.62	5552.14	5504.82
	BIC	6289.76	5784.92	5798.70	5618.10	5583.82

Note: Cells show the unstandardized estimates and their standard deviations in brackets. * *p* < 0.05, *** *p* < 0.001, ns = not significant.

## Data Availability

The datasets generated and analyzed during the current study are not publicly available but are available from the corresponding author on reasonable request.
